# ASAP1 Expression in Invasive Breast Cancer and Its Prognostic Role

**DOI:** 10.3390/ijms241814355

**Published:** 2023-09-20

**Authors:** Hosub Park, Hwangkyu Son, Hyebin Cha, Kihyuk Song, Seongsik Bang, Seungyun Jee, Hyunsung Kim, Jaekyung Myung, Su-Jin Shin, Chihwan Cha, Min Sung Chung, Seungsam Paik

**Affiliations:** 1 Department of Pathology, Hanyang University Hospital, Hanyang University College of Medicine, Seoul 04763, Republic of Korea; 2 Department of Pathology, Asan Medical Center, University of Ulsan College of Medicine, Seoul 06273, Republic of Korea; 3 Department of Pathology, Gangnam Severance Hospital, Yonsei University College of Medicine, Seoul 06273, Republic of Korea; 4 Department of Surgery, Hanyang University Hospital, Hanyang University College of Medicine, Seoul 04763, Republic of Korea

**Keywords:** ArfGAP with SH3 domain, ankyrin repeat, PH domain 1, invasive breast carcinoma, prognosis

## Abstract

Breast cancer is a major global health burden with high morbidity and mortality rates. Previous studies have reported that increased expression of ASAP1 is associated with poor prognosis in various types of cancer. This study was conducted on 452 breast cancer patients who underwent surgery at Hanyang University Hospital, Seoul, South Korea. Data on clinicopathological characteristics including molecular pathologic markers were collected. Immunohistochemical staining of ASAP1 expression level were used to classify patients into high and low groups. In total, 452 cases low ASAP1 expression group was associated with significantly worse recurrence-free survival (*p* = 0.029). In ER-positive cases (*n* = 280), the low ASAP1 expression group was associated with significantly worse overall survival (*p* = 0.039) and recurrence-free survival (*p* = 0.029). In multivariate cox analysis, low ASAP1 expression was an independent significant predictor of poor recurrence-free survival in the overall patient group (hazard ratio = 2.566, *p* = 0.002) and ER-positive cases (hazard ratio = 4.046, *p* = 0.002). In the analysis of the TCGA dataset, the low-expression group of ASAP1 protein demonstrated a significantly poorer progression-free survival (*p* = 0.005). This study reports that low ASAP1 expression was associated with worse recurrence-free survival in invasive breast cancer.

## 1. Introduction

Breast cancer is a major global health burden. Breast cancer is the most common cancer; a total of 2.26 million new cases of breast cancer were reported worldwide in 2020, representing 11.7% of all cancer cases. Breast cancer caused 680,000 deaths worldwide in 2020, accounting for 6.9% of all cancer deaths; it ranks as the fifth leading cause of death [1]. In South Korea, breast cancer is the fifth most common cancer and the seventh leading cause of death. The incidence rate of breast cancer was 48.5 cases per 100,000 individuals in 2020, and the mortality rate was 5.3 deaths per 100,000 population [2].

The most commonly used molecular pathological classification of breast cancer is based on surrogate markers and was introduced in the 2013 St. Gallen guidelines [3]. Immunohistochemical (IHC) staining and in situ hybridization (ISH) are used to assess the expression of estrogen receptor (ER), progesterone receptor (PR), human epidermal growth factor receptor-2 (HER2), and Ki-67 labelling index. In this classification, breast cancer is divided into subtypes “luminal A-like”, “luminal B-like”, “HER2 positive (non-luminal)”, and “triple-negative”. The luminal A-like subtype and luminal B-like subtype are both ER-positive, while a subset of the luminal B-like subtype and the HER2-positive (non-luminal) subtype are HER2-positive. The triple-negative subtype is negative for both ER and HER2.

ER and HER2 status is important in determining the treatment approach for early-stage breast cancer. ER-positive breast cancers are treated with hormone therapy [4]. Compared with the survival of ER-negative breast cancer, the 5-year survival rate is generally favorable, but late recurrence can occur several years after the initial diagnosis. Therefore, additional chemotherapy may provide a benefit in terms of prognosis [5,6]. In ER-positive breast cancer patients, in addition to stage and HER2 status evaluation, gene expression profile signature tests such as MammaPrint or Oncotype DX are conducted to determine the need for additional chemotherapy [4,7].

ArfGAP with SH3 domain, ankyrin repeat, and PH domain 1 (ASAP1) protein modulates various functions in cells such as receptor recycling, vesicle formation, and cell motility by interacting with a diverse range of proteins in the ADP-ribosylation factor (ARF) family [8]. Previous studies have reported that the increased expression of ASAP1 promotes invasiveness and metastasis in cancer [9,10,11,12,13,14,15,16,17]. The majority of these studies also have reported that the increased expression of ASAP1 is associated with poor prognosis in various types of cancer. Increased ASAP1 expression enhanced tumor cell invasiveness and metastasis in colorectal cancer [9] and was associated with metastasis in prostate cancer [10]. Increased expression of ASAP1 was correlated with poor prognosis in head and neck squamous cell carcinoma [11,12], hepatocellular carcinoma [13], gastric cancer [14], epithelial ovarian cancer [15], clear cell renal cell carcinoma [16], and pancreatic cancer [17].

However, recent studies have presented contrasting findings. In hepatoblastoma, the loss of ASAP1 and EGFR expression was observed in histologically poor differentiated cases, and this trend was more pronounced in metastatic and unresectable cases [18]. In a luminal breast cancer mouse model, loss of ASAP1 expression increased AKT signal activation and tumor cell invasion. The ASAP1 knockout mice model showed significantly faster tumor growth and more frequent lung metastasis compared with the wild-type model [19]. These contradictory results highlight the need for further research to fully understand the role of ASAP1 in breast cancer. Thus, the role of ASAP1 in cancer progression and metastasis warrants further investigation.

This study investigated the role of ASAP1 protein in invasive breast cancer. The expression level of ASAP1 protein was measured in surgical specimens of primary invasive breast cancer cases by immunohistochemical staining. ASAP1 protein expression was compared with clinicopathological parameters and patient outcomes.

## 2. Results

### 2.1. Baseline Characteristics of the Patients

The baseline characteristics of the 452 breast cancer patients included in this study are summarized in Table 1. The mean age of the patients was 52.8 ± 10.8 years, with a range of 27 to 83 years. The mean tumor size was 2.6 ± 1.8 cm, with a range of 0.12 to 16 cm. According to the Nottingham histologic grading system [20], 99 cases (21.90%) were G1, 200 cases (44.25%) were G2, and 153 cases (33.85%) were G3. The majority of patients had either T1 or T2 stage tumors, with 202 cases (44.69%) and 214 cases (47.34%), respectively. Most patients had N0 stage disease (285 cases, 63.05%). Only eight cases showed a distant metastasis status. The majority of tumors were ER-positive (280 cases, 61.95%), while HER2-positivity was observed in 143 cases (31.64%). The distribution of molecular subtypes was as follows: luminal A-like, 187 cases (41.37%); luminal B-like, 93 cases (20.58%); HER2 positive (non-luminal), 76 cases (16.81%); and triple-negative, 96 cases (21.24%). In terms of ASAP1 expression status, 176 cases (38.94%) showed high expression, while 276 cases (61.06%) exhibited low expression. The recurrence rate was 16.59%, and the survival rate was 89.60%.

### 2.2. ASAP1 Expression and Its Correlations with Clinicopathological Characteristics in Total Cases

**Figure 1:**
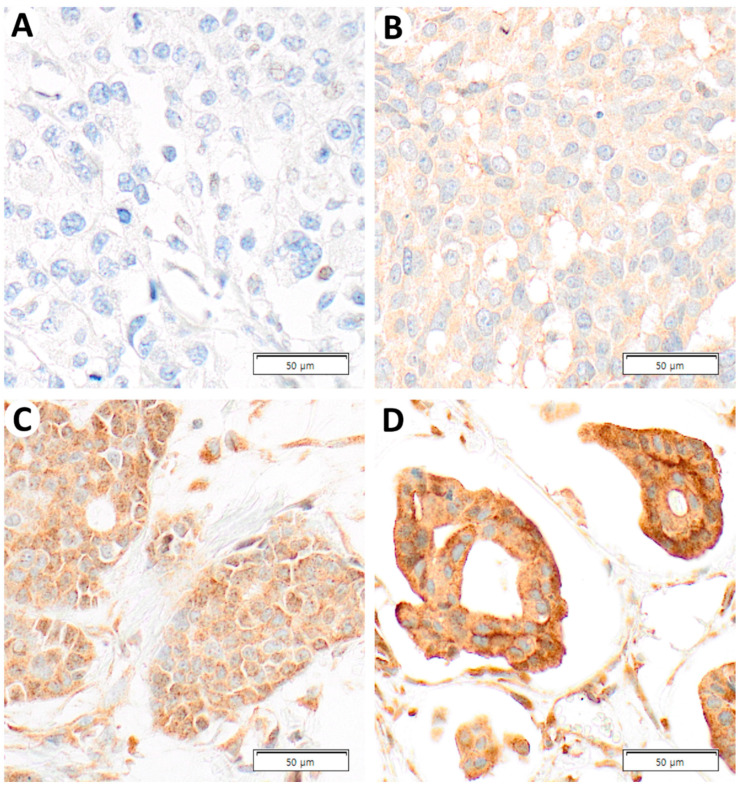
Representative photomicrographs of ASAP1 Immunohistochemical (IHC) staining on the cancer tissue. (**A**) Negative (×400), (**B**) weak (×400), (**C**) moderate (×400), and (**D**) strong (×400).

Of 452 breast cancer cases, 176 cases showed high ASAP1 expression, and 276 cases showed low ASAP1 expression. Representative images depicting staining intensity are shown in Figure 1.

The correlations between ASAP1 expression and clinicopathological characteristics in 452 breast cancer patients are summarized in Supplementary Table S1. There was no significant association between ASAP1 expression and age, tumor size, histological grade, T stage, N stage, distant metastasis, ER status, HER2 status, Ki-67 index, or molecular subtypes.

### 2.3. Prognostic Value of ASAP1 Expression in Total Cases

**Figure 2:**
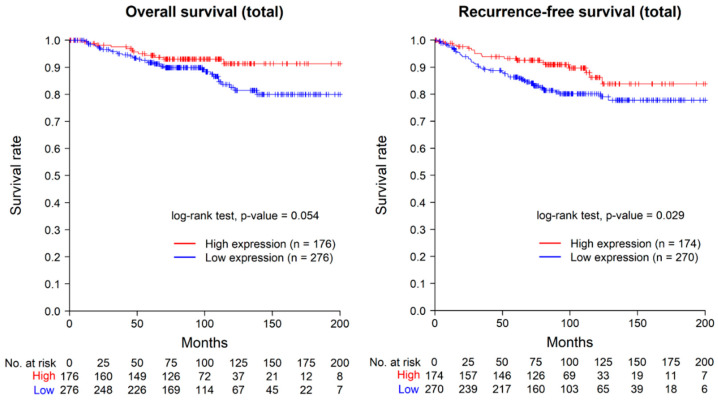
Kaplan–Meier curves for overall survival (**left**) and recurrence-free survival (**right**) in total. Eight cases with distant metastasis at the time of diagnosis were excluded in the analysis of recurrence-free survival.

The Kaplan–Meier graphs for overall survival and recurrence-free survival in all 452 patients are presented in Figure 2. In the Kaplan–Meier analysis of overall survival, a trend towards a poorer prognosis was observed in the ASAP1-low-expression group; however, it was not significant statistically (log-rank test, *p* = 0.054). For recurrence-free survival, the ASAP1-low-expression group demonstrated a statistically significant poorer prognosis (log-rank test, *p* = 0.029). Eight cases with distant metastasis at the time of diagnosis were excluded from the recurrence-free survival analysis.

When Kaplan–Meier survival analysis was applied to the molecular subtypes, the low ASAP1 expression group in the luminal B-like subtype showed significantly poorer prognosis in overall survival (log-rank test, *p* = 0.023) (Supplementary Figure S1). In the analysis of recurrence-free survival, the low ASAP1 expression group in the Luminal A-like subtype and HER2 positive (non-luminal) subtype exhibited a tendency to have poorer prognosis; however, they were not statistically significant (log-rank test, *p* = 0.065 and 0.063, respectively) (Supplementary Figure S2).

The results of univariate and multivariate Cox regression analyses for recurrence-free survival in the 452 cases are summarized in Table 2. In univariate analysis, size (*p* < 0.001), T stage (*p* < 0.001), histological grade (G2 vs. G1, *p* = 0.022 and G3 vs. G1, *p* = 0.002), N stage (N1, N2, N3 vs. N0, *p* < 0.001), and low ASAP1 expression (*p* < 0.031) were significantly associated with worse recurrence-free survival. In multivariate analysis, low ASAP1 expression was an independent significant predictor of poor recurrence-free survival (hazard ratio = 2.566, *p* = 0.002).

### 2.4. Correlations between ASAP1 Expression and Clinicopathological Characteristics in ER-Positive Cases

The correlation between ASAP1 expression and clinicopathological characteristics in 280 ER-positive breast cancer patients is summarized in Supplementary Table S2. Among the ER-positive cases, 107 cases showed high ASAP1 expression, and 173 cases showed low ASAP1 expression. The Ki-67 index of the tumors showed a statistically significant difference between ASAP1 expression groups (*p* = 0.036). However, there was no significant association between ASAP1 expression and age, tumor size, histological grade, T stage, N stage, distant metastasis, ER status, HER2 status, or molecular subtypes.

### 2.5. Prognostic Value of ASAP1 Expression in ER-Positive Cases

**Figure 3:**
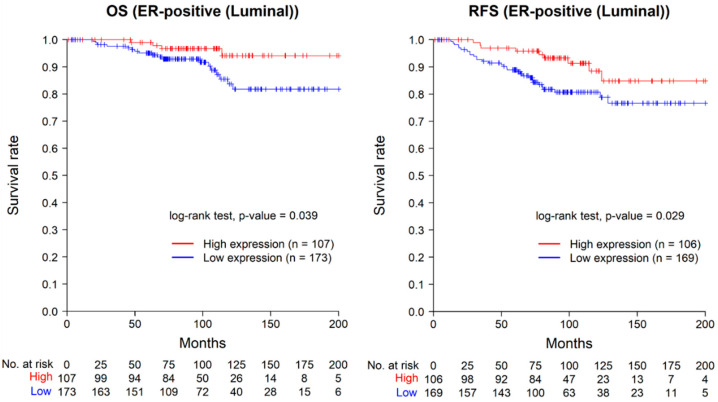
Kaplan–Meier curves for overall survival (**left**) and recurrence-free survival (**right**) in ER-positive cases. Five cases with distant metastasis at the time of diagnosis were excluded in the analysis of recurrence-free survival. OS, overall survival; RFS, recurrence-free survival.

The Kaplan–Meier graphs for overall survival and recurrence-free survival in 280 ER-positive breast cancer patients are shown in Figure 3. The low-expression group for ASAP1 showed significantly worse prognosis in both overall survival and recurrence-free survival (log-rank test, *p* = 0.039 and *p* = 0.029, respectively). Five cases with distant metastasis at the time of diagnosis were excluded from the recurrence-free survival analysis.

The results of univariate and multivariate Cox regression analyses for recurrence-free survival in 280 ER-positive cases are summarized in Table 3. In univariate analysis, size (*p* < 0.001), histological grade (G2 vs. G1, *p* = 0.033 and G3 vs. G1, *p* = 0.001), N stage (N1 vs. N0, *p* < 0.001 and N2 vs. N0, *p* = 0.011 and N3 vs. N0, *p* < 0.001), and low ASAP1 expression (*p* = 0.033) were significantly associated with worse recurrence-free survival. In multivariate analysis, low ASAP1 expression was an independently significant predictor of poor recurrence-free survival (hazard ratio = 4.046, *p* = 0.002).

### 2.6. Results of Public Dataset Analysis

**Figure 4:**
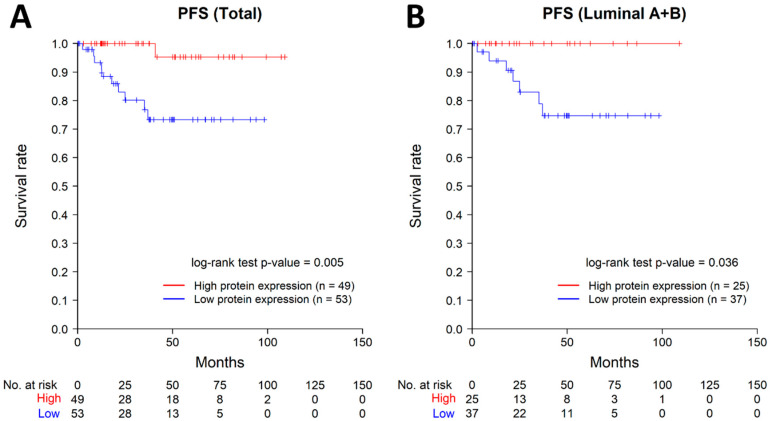
Kaplan–Meier curves for progression-free survival on protein expression level with TCGA PanCancer Atlas data. (**A**) Progression-free survival in total cases (*n* = 102), (**B**) progression-free survival in ER-positive cases (*n* = 62). PFS, progression-free survival.

Additional Kaplan–Meier survival analysis for validation was performed using breast cancer data from the TCGA PanCancer Atlas. Kaplan–Meier analysis revealed that the low-expression group for ASAP1 protein exhibited significantly worse progression-free survival in total cases (*p* = 0.005) and ER-positive subtypes (*p* = 0.036), which include luminal A-like and B-like subtypes (Figure 4).

## 3. Discussion

In this study, the expression level of ASAP1 protein was investigated in 452 surgical specimens of primary invasive breast cancer using immunohistochemical staining and the relationship between ASAP1 expression and patient outcomes was evaluated. There was no significant association between ASAP1 expression and several clinicopathological features including age, tumor size, histological grade, T stage, N stage, ER status, PR status or molecular subtypes. In Kaplan–Meier survival analyses, low ASAP1 expression was significantly associated with poor recurrence-free survival in total cases and was significantly associated with poor overall survival and recurrence-free survival in ER-positive cases. In Cox regression analyses for recurrence-free survival in both total cases and ER-positive cases, low ASAP1 expression was significantly associated with worse recurrence-free survival in univariate analysis. In multivariate analysis, low ASAP1 expression was an independent significant predictor of poor recurrence-free survival.

Interestingly, this study demonstrated that low ASAP1 expression was associated with a worse prognosis in terms of both overall survival and recurrence-free survival. This finding is contrary to the results of previous studies suggesting that high expression level of ASAP1 correlates with poor prognosis. Although the observed trend towards poorer overall survival for the low ASAP1 expression group (*p* = 0.054) was not statistically significant, recurrence-free survival was significantly worse in the low ASAP1 expression group (*p* = 0.029) in the analysis of total cases. In the analysis of ER-positive cases, low ASAP1 expression was significantly associated with poor overall survival (*p* = 0.039) and recurrence-free survival (*p* = 0.029). These findings suggest that low ASAP1 expression may be associated with a higher risk of recurrence in breast cancer patients.

The results of univariate and multivariate Cox regression analyses for recurrence-free survival further support the hypothesis that low ASAP1 expression could be a significant predictor of poor recurrence-free survival. The results of multivariate Cox regression analysis demonstrated that low expression of ASAP1 protein independently conferred a 2.566-fold-higher risk of recurrence in total cases. When ER-positive cases were analyzed, similar findings were observed. Multivariate Cox regression analysis revealed that low expression of ASAP1 protein was independently correlated with a 4.046-fold-higher risk of recurrence.

This study also identified the difference in overall survival or recurrence-free survival between molecular subtypes according to the ASAP1 expression. In overall survival analysis, luminal B-like patients showed significantly worse overall survival in the low ASAP1 expression group (*p* = 0.023). In recurrence-free survival analysis, Luminal A-like patients and HER2-positive patients showed a tendency to have worse prognosis, which were not statistically significant (log-rank test, *p* = 0.065 and 0.063, respectively). These findings suggest that the impact of ASAP1 expression may be influenced by the molecular subtype of the tumor.

Previous studies have reported that the ASAP1 protein is overexpressed in a variety of cancers, promoting invasion and metastasis, and is associated with poor prognosis. Muller et al. reported that higher ASAP1 expression was found in colorectal adenocarcinomas with metastasis [9]. Lin et al. reported that ASAP1 expression was significantly higher in metastatic prostate cancer [10]. Li et al. reported that patients with higher ASAP1 mRNA expression levels showed significantly poorer overall survival [11]. Sato et al. reported that high expression of ASAP1 protein in head and neck squamous cell carcinoma is associated with poor prognosis [12]. Bang et al. reported that high ASAP1 expression is associated with poor prognosis in hepatocellular carcinoma [13]. Lou et al. reported that higher expression of ASAP1 and FAK is associated with higher stage, poor differentiation, lymph node metastasis, and worse prognosis in the gastric cancer [14]. Hou et al. showed that high ASAP1 expression in epithelial ovarian cancer is significantly associated with pelvic metastasis and worse prognosis [15]. Hashimoto et al. reported that increased ASAP1 expression is associated with poor prognosis in clear cell renal cell carcinoma [16]. Tsutaho et al. reported that increased ASAP1 protein expression is associated with increased fibrosis and PD-L1 expression in pancreatic cancer [17].

Similar results have been reported in breast cancer. Onodera et al. compared ASAP1 protein expression in 28 cases of ductal carcinoma in situ (DCIS) and invasive ductal carcinoma, reporting higher expression in invasive duct al carcinoma compared to DCIS [20]. Kinoshita et al. analyzed the surgical specimen of 20 patients who had local recurrence. They reported a significant association between high expression of GEP100 and ASAP1 protein in tumor tissue and local recurrence [21]; He et al. reported that copy number gain and mRNA overexpression of ASAP1 occur more frequently in triple-negative breast cancer (TNBC) compared to other breast cancer subtypes. In their study, they showed that higher mRNA expression levels of ASAP1 have significantly worse prognosis in 257 TNBC cases from METABRIC data. However, their study also showed that the prognostic difference was not statistically significant in 2519 cases of ER-positive breast cancer, unlike in triple-negative breast cancer [22].

Recent studies have reported contradictory results. Ranganathan et al. analyzed protein expression of EGFR and ASAP1 through immunohistochemical staining in 60 cases of hepatoblastoma. The authors observed that the expression of ASAP1 and EGFR was decreased in poorly differentiated cases. They also reported that this trend is stronger in cases with metastasis or unresectable tumors [18]. Schreiber et al. reported that the loss of ASAP1 expression was associated with increased AKT signal activation and tumor cell invasion in their study using a mouse model for luminal breast cancer. Additionally, the authors compared wild-type models with ASAP1 knockout models, and the ASAP1 knockout models exhibited significantly faster tumor growth, more lung metastasis, and higher invasive capacity of tumor cells [19]. These studies have shown contradictory findings regarding the association between ASAP1 expression and prognosis in breast cancer. Our results support the findings reported by Schreiber et al.

Furthermore, the result of TCGA data analysis in the current study showed similar findings in the luminal subtypes. The low ASAP1 protein expression group exhibited significantly worse progression-free survival in all cases (*p* = 0.005) and ER-positive cases (*p* = 0.036), which include the luminal A-like and B-like subtypes. However, in triple-negative subtype, the expression levels of both mRNA and protein have no significant difference in progression-free survival (Supplementary Figure S3). These findings suggest the possibility that the function of ASAP1 may vary depending on the context.

This study suggests the potential utility of ASAP1 as a predictive factor for poor prognosis in ER-positive breast cancer. This highlights the significance of ASAP1 as a prognostic indicator that can help predict outcomes in this specific subset of breast cancer patients. Additionally, contrary to most previous findings, this study indicates the possibility that ASAP1 may act as a tumor suppressor in specific contexts. However, the mechanism of ASAP1 action in ER-positive breast cancer is poorly understood. Future studies should explore these contexts and interactions with other molecular pathways, particularly those associated with estrogen receptor. The clinical implications of targeting ASAP1 as a therapeutic strategy, such as developing specific inhibitors or exploring its potential as a predictive biomarker for treatment response, would be valuable areas of investigation. Moreover, expanding research to larger patient cohorts and diverse breast cancer subtypes could provide more comprehensive insight into the clinical relevance and generalizability of ASAP1 as a prognostic and therapeutic target.

There are some limitations to this study. Since, the tissue specificity of ASAP1 has not yet been established, the authors could not apply separate negative and positive controls. Instead, the authors employed normal breast tissue adjacent to the tumor tissue as an internal control. The non-neoplastic mammary lobules adjacent to the cancer demonstrated negative to weak, heterogeneous positivity on ASAP1 staining. A representative image of the internal control tissue is in Supplementary Figure S4. The absence of separate negative and positive controls, as well as secondary antibody controls, could be considered as a limitation of this study. However, this study focused on analyzing the relative expression levels of ASAP among cancer tissues and employed an internal control. Consequently, the authors estimate that the observed results are not significantly biased due to these limitations. Another limitation of this study is that it is a retrospective study, and the included cases were collected from a single center. Therefore, despite of large number of cases, there could be selective bias. Moreover, in this study, ASAP1 expression was measured only at the protein level. Analysis of mRNA or miRNA expression would be necessary in future studies.

In conclusion, the expression level of ASAP1 protein was investigated in 452 surgical specimens of primary invasive breast cancer. Low ASAP1 protein expression was associated with worse recurrence-free survival in invasive breast cancer. ASAP1 expression may serve as a potential prognostic biomarker for personalized treatment decisions in breast cancer patients. Further research is warranted to elucidate the underlying mechanisms and validate the clinical utility of ASAP1 in breast cancer prognosis.

## 4. Materials and Methods

### 4.1. Patients and Tissue Samples

This study was a retrospective analysis of 452 female breast cancer patients who underwent surgery at Hanyang University Hospital between February 2003 and January 2017. The inclusion criteria for this study were pathologically confirmed invasive breast carcinoma and available paraffin-embedded tissue samples. Patients with non-invasive carcinoma or insufficient medical records were excluded. Clinicopathological characteristics including age, tumor size, histological grade, TNM stage, and molecular subtype were collected from medical records and pathology reports. The histological grade in the pathological reports was recorded by the Nottingham Grading System [23]. This study was approved by the Institutional Review Board of the Hanyang University Hospital (HYUH 2021-12-014-002), and the need for informed consent was waived.

### 4.2. Tissue Microarray (TMA) Construction

Archived histologic slides from the included cases were reviewed. The most representative non-necrotic central portion of the tumor was selected by light microscopy. From the paraffin blocks of these representative tumor section, cylindrical tissue cores 3 mm in diameter were extracted and transferred to the recipient block (Unitma, Seoul, Republic of Korea) by a manual tissue microarrayer (Unitma, Seoul, Republic of Korea). Each TMA block was comprised of 6 × 5 samples.

### 4.3. Immunohistochemical (IHC) Staining

IHC staining for ER, PR, HER2, and ASAP1 was performed on 4 μm thick TMA sections using the Benchmark XT automated staining system (Ventana Medical Systems, Tucson, AZ, USA). The primary antibodies for ER, PR, and HER2 were as follows: ER (SP1) rabbit monoclonal primary antibody (Roche, Basel, Switzerland), PR (1E2) rabbit monoclonal primary antibody (Roche), HER-2/neu (4B5) rabbit monoclonal primary antibody (Roche), and anti-ASAP1/DDEF1 antibody (7B12) (ab125729, Abcam, Cambridge, UK; diluted 1:200). Heat-induced epitope retrieval was performed with CC1 Tris-EDTA buffer (Ventana Medical Systems, Tucson, AZ, USA) at 100 °C for 80 min. Detection of primary antibody was performed with the OptiView DAB IHC Detection Kit (Ventana Medical Systems). IHC staining for Ki-67 was performed on 4 μm thick TMA sections using the Bond-Max automated immunostainer (Leica BioSystems, Newcastle, UK). Monoclonal (MIB-1) Ki-67 antibody (M7240, Dako, Santa Clara, CA, USA; diluted 1:100) was also used for IHC. Heat-induced antigen retrieval was performed with Bond epitope retrieval solution (Leica BioSystems) after deparaffinization. Detection of primary antigen was performed using the Bond Polymer Refine Detection kit (Leica BioSystems) and 3,3’-diaminobenzidine tetrahydrochloride as a chromogen.

### 4.4. Interpretation of IHC Staining

Immunohistochemical staining for ASAP1 was interpreted applying the H-score method [24]. The staining intensity in tumor cell cytoplasm was assessed as an intensity score of 0, 1, 2, 3, representing negative, weak, moderate, and strong intensity, respectively. The percentage of tumor cells stained at each intensity was evaluated as a proportion score ranging from 0 to 100 by 10 points increments. The H-score, ranging from 0 to 300, was calculated by sum of the values that multiplied each intensity scores by its proportional score. The calculation of the H-score is in the equation below, where ‘*i*’ represents each intensity, and 1, 2, 3 correspond to weak, moderate, and strong, respectively.

$$H-score=\sum_{i=1,2,3}\left({intensity\;score}_{i}\times {intensity\;score}_{i}\right)$$

The cut-off point for the H-score was determined as 10 based on the highest Youden index, in the receiver operating characteristic (ROC) curve for overall survival. The Youden index is one of the frequently used methods to choose an optimal cut-off point of a diagnostic marker. The Youden index of a certain threshold ($J_{\left(c\right)}$) is calculated by sensitivity (${Sensitivity}_{\left(c\right)})$ and specificity (${Specificity}_{\left(c\right)})$ in that certain threshold(c), as below [25].

$$J_{\left(c\right)}={Sensitivity}_{\left(c\right)}+{Specificity}_{\left(c\right)}-1$$

As a result, Cases with a H-score of 10 or lower were classified as the low-expression group, while cases with a H-score more than 10 were classified as the high-expression group. ER, PR, and HER2 status was classified as positive or negative according to the ASCO/CAP guidelines [26,27]. Although there is no international consensus on the cut-off point for Ki-67, many studies use a range of 10% to 20% as the cut-off [28]. In this study, the Ki-67 index with values equal or less than 10% categorized as low, values more than 10% but equal or less than 20% as intermediate, and values more than 20% as high.

### 4.5. The Cancer Genome Atlas (TCGA) PanCancer Atlas Data

The TCGA PanCancer Atlas data for invasive breast carcinoma was obtained through the CbioPortal [29]. Protein expression data was obtained from Reverse Phase Protein Array (RPPA) data and used the Z scores. The value of the highest Youden index was used as the cut-off point to categorize samples into low- and high-expression groups. Recurrence-free survival analysis was performed.

### 4.6. Statistical Analysis

Statistical analysis was performed using R version 4.1.1 and R studio 2021.09.0 + 351 “Ghost Orchid” Release. The association between clinicopathological features and ASAP1 expression groups was analyzed using Student’s *t*-test, Chi-squared test, and Fisher’s exact test. Survival analysis was conducted using the Kaplan–Meier method with log-rank test and Cox proportional hazards model. A *p*-value of less than 0.05 was considered statistically significant.

## Figures and Tables

**Table 1:** Baseline characteristics of breast cancer patients (*n* = 452).

Clinicopathological Characteristics	Value
Age (years, mean ± SD)	52.8 ± 10.8 (27–83)
Size (cm, mean ± SD)	2.6 ± 1.8 (0.12–16)
Histological grade *	
G1	99 (21.90%)
G2	200 (44.25%)
G3	153 (33.85%)
T stage ^†^	
T1	202 (44.69%)
T2	214 (47.34%)
T3	23 (5.09%)
T4	13 (2.88%)
N stage ^†^	
N0	285 (63.05%)
N1	98 (21.68%)
N2	38 (8.41%)
N3	31 (6.86%)
Distant metastasis	
Negative	444 (98.23%)
Positive	8 (1.77%)
ER status	
Negative	172 (38.05%)
Positive	280 (61.95%)
HER2 status	
Negative	309 (68.36%)
Positive	143 (31.64%)
Ki-67 index	
Low (≤10%)	279 (61.725%)
Intermediate (>10%, ≤20%)	53 (11.725%)
High (>20%)	120 (26.55%)
Molecular subtype ^‡^	
Luminal A-like	187 (41.37%)
Luminal B-like	93 (20.58%)
HER2 (non-luminal)	76 (16.81%)
Triple-negative	96 (21.24%)
ASAP1 expression	
High	176 (38.94%)
Low	276 (61.06%)
Recurrence	
Negative	377 (83.41%)
Positive	75 (16.59%)
Survival	
Survive	405 (89.60%)
Dead	47 (10.40%)

* Nottingham Grading System, ^†^; AJCC cancer staging manual, 8th edition, ^‡^; St Gallen classification, 2013; ER: estrogen receptor; HER2: human epidermal growth factor receptor 2; ASAP1: ArfGAP with SH3 domain; ankyrin repeat and PH domain 1.

**Table 2:** Univariate and multivariate Cox regression analyses for recurrence-free survival in total cases.

		Univariate Analysis			Multivariate Analysis		
		HR	95% CI	*p*-Value	HR	95% CI	*p*-Value
Age	(per year)	0.992	0.969–1.016	0.503	1.007	0.984–1.031	0.551
Size	(per cm)	1.24	1.144–1.344	<0.001	1.103	0.981–1.24	0.102
T stage	T4 (vs. T1,2,3)	7.792	3.357–18.089	<0.001	4.579	1.493–14.044	0.008
Histological grade	G2 (vs. G1)	3.015	1.17–7.773	0.022	2.393	0.91–6.292	0.077
	G3 (vs. G1)	4.547	1.771–11.67	0.002	3.106	1.102–8.753	0.032
N stage	N1 (vs. N0)	3.337	1.86–5.988	<0.001	2.935	1.593–5.407	0.001
	N2 (vs. N0)	4.013	1.945–8.278	<0.001	4.155	1.958–8.82	<0.001
	N3 (vs. N0)	5.189	2.515–10.708	<0.001	4.454	2.05–9.677	<0.001
ER status	Positive (vs. Negative)	0.786	0.484–1.278	0.332	0.95	0.54–1.671	0.858
HER2 status	Positive (vs. Negative)	1.305	0.795–2.142	0.293	1.114	0.669–1.856	0.678
Ki-67 index	Intermediate (vs. Low)	0.719	0.305–1.697	0.452	0.843	0.352–2.018	0.702
	High (vs. Low)	1.206	0.711–2.046	0.487	0.868	0.486–1.551	0.632
ASAP1 expression	Low (vs. High)	1.813	1.056–3.113	0.031	2.566	1.429–4.607	0.002

HR: hazard ratio; CI: confidence interval; ER: estrogen receptor; HER2: human epidermal growth factor receptor 2; ASAP1: ArfGAP with SH3 domain, ankyrin repeat and PH domain 1.

**Table 3:** Univariate and multivariate Cox regression analyses for recurrence-free survival in ER-positive cases.

		Univariate Analysis			Multivariate Analysis		
		HR	95% CI	*p*-Value	HR	95% CI	*p*-Value
Age	(per year)	0.993	0.963–1.024	0.643	1.015	0.984–1.048	0.345
Size	(per cm)	1.319	1.159–1.502	<0.001	1.238	1.03–1.488	0.023
T stage	T4 (vs. T1,2,3)	3.888	0.531–28.477	0.181	2.458	0.236–25.626	0.452
Histological grade	G2 (vs. G1)	3.195	1.101–9.275	0.033	2.35	0.783–7.054	0.128
	G3 (vs. G1)	6.48	2.113–19.878	0.001	6.53	1.839–23.182	0.004
N stage	N1 (vs. N0)	4.657	2.132–10.174	<0.001	3.416	1.469–7.945	0.004
	N2 (vs. N0)	4.015	1.372–11.749	0.011	2.889	0.873–9.565	0.082
	N3 (vs. N0)	6.581	2.504–17.298	<0.001	4.133	1.428–11.966	0.009
HER2 status	Positive (vs. Negative)	0.981	0.477–2.016	0.957	0.596	0.259–1.371	0.224
Ki-67 index	Intermediate (vs. Low)	0.977	0.404–2.362	0.959	1.139	0.453–2.862	0.782
	High (vs. Low)	0.806	0.311–2.089	0.658	0.257	0.08–0.831	0.023
ASAP1 expression	Low (vs. High)	2.246	1.065–4.734	0.033	4.046	1.644–9.957	0.002

HR: hazard ratio; CI: confidence interval; HER2: human epidermal growth factor receptor 2; ASAP1: ArfGAP with SH3 domain, ankyrin repeat and PH domain 1.

## Data Availability

The TCGA PanCancer Atlas data for invasive breast carcinoma was obtained through the CbioPortal. This data can be found here [https://www.cbioportal.org/study/summary?id=brca_tcga_pan_can_atlas_2018]. The database from Hanyang University Hospital is not publicly available, because the public release of the data is not approved by the Institutional Review Board.

## Supplementary Materials

The supporting information can be downloaded at: https://www.mdpi.com/article/10.3390/ijms241814355/s1.
