# Supplementary material for: ASAP1 Expression in Invasive Breast Cancer and Its Prognostic Role
Source: Int J Mol Sci. 2023 Sep 20;24(18):14355. doi: 10.3390/ijms241814355 (PMC10532164; doi:10.3390/ijms241814355)
Supplement: Supplementary file 1 [file ijms-24-14355-s001.zip › ijms-2587741-supplementary.pdf]

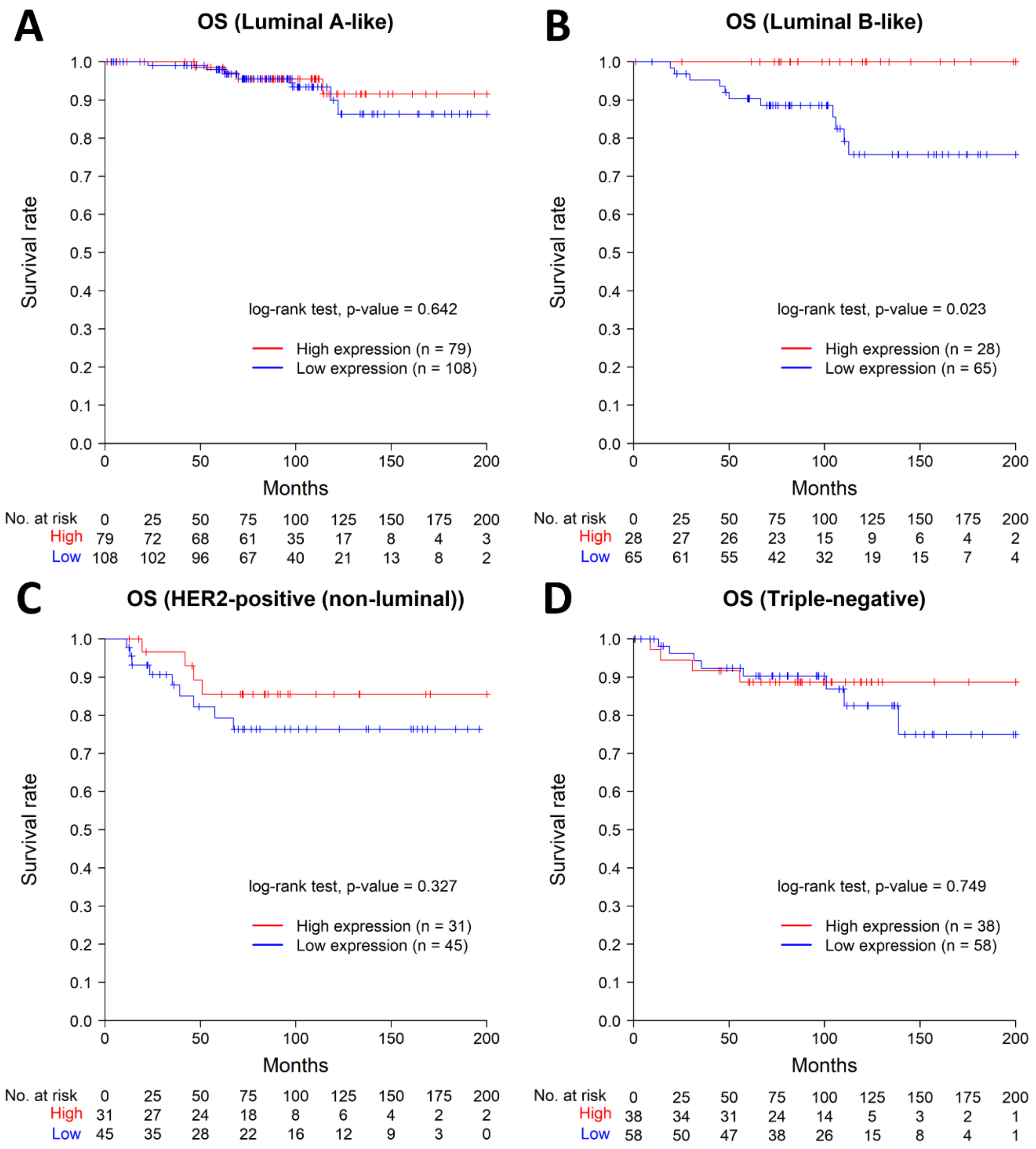

Figure S1. Kaplan–Meier overall survival curves according to the molecular subtypes. A: Overall survival for luminal A-like subtype, B: overall survival for luminal B-like subtype, C: overall survival for HER2-positive (non-luminal) subtype, and D: overall survival for triple-negative subtype. OS, overall survival.

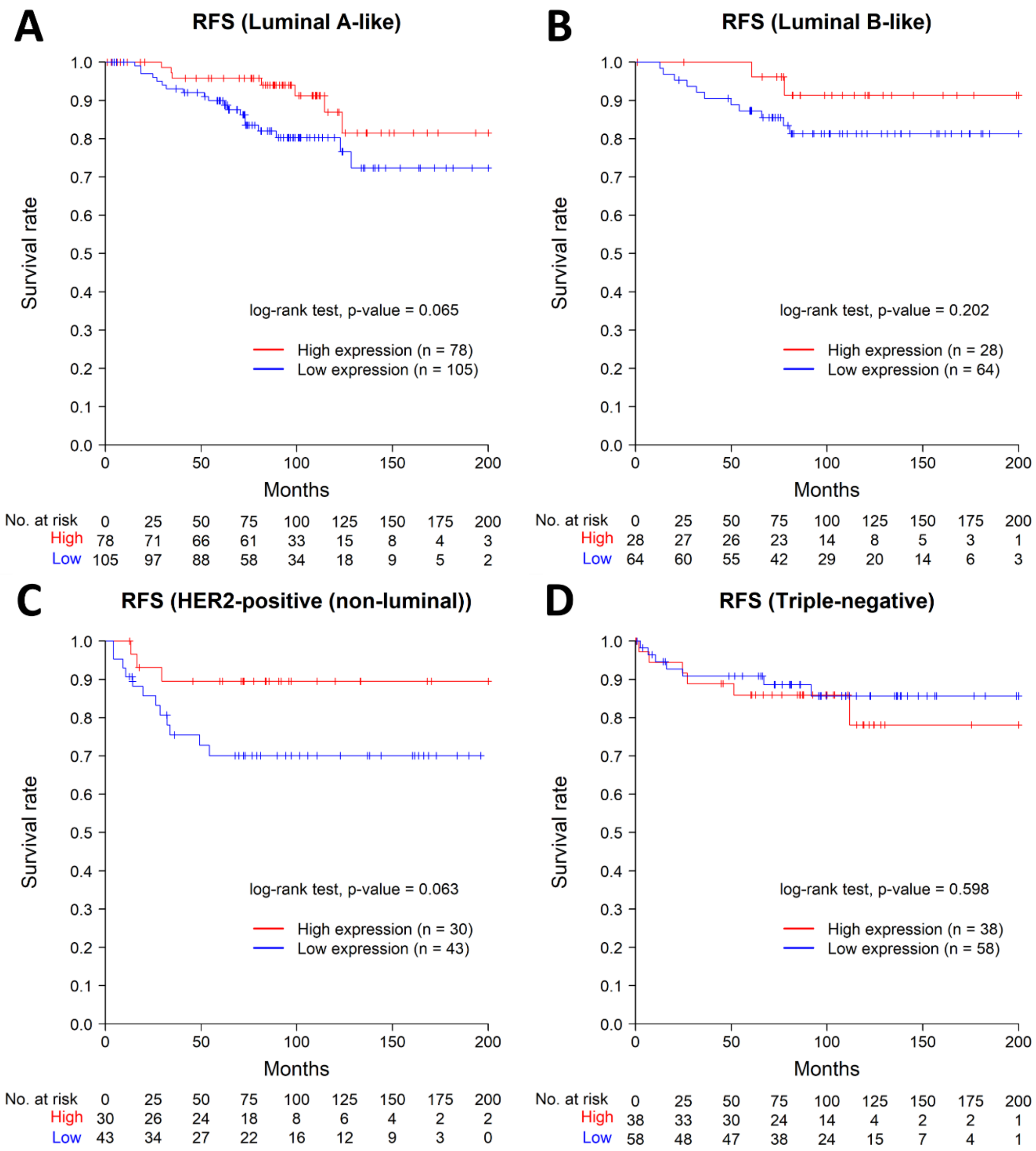

Figure S2. Kaplan–Meier recurrence-free survival curves according to the molecular subtype. A: Recurrence-free survival for luminal A-like subtype, B: recurrence-free survival for luminal B-like subtype, C: recurrence-free survival for HER2-positive (non-luminal) subtype, and D: recurrence-free survival for triple-negative subtype. RFS, recurrence-free survival.

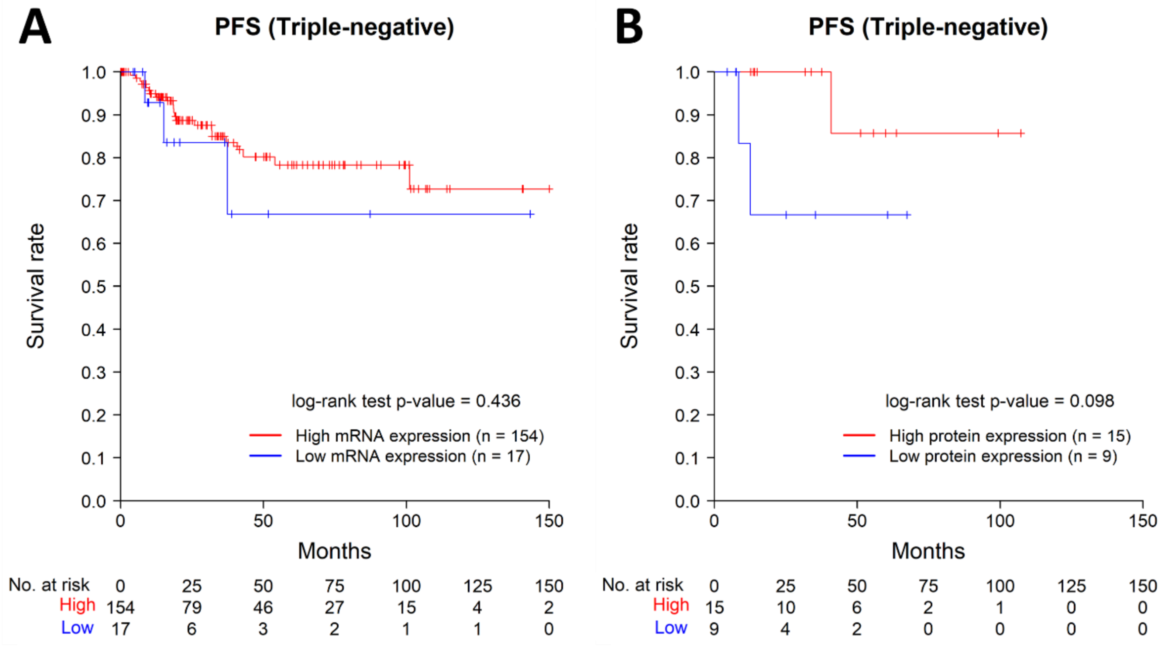

Figure S3. Kaplan–Meier curves for recurrence-free survival in Triple-negative subtype with TCGA PanCancer Atlas data. A: on mRNA expression level, B: on protein expression level. PFS, progression-free survival

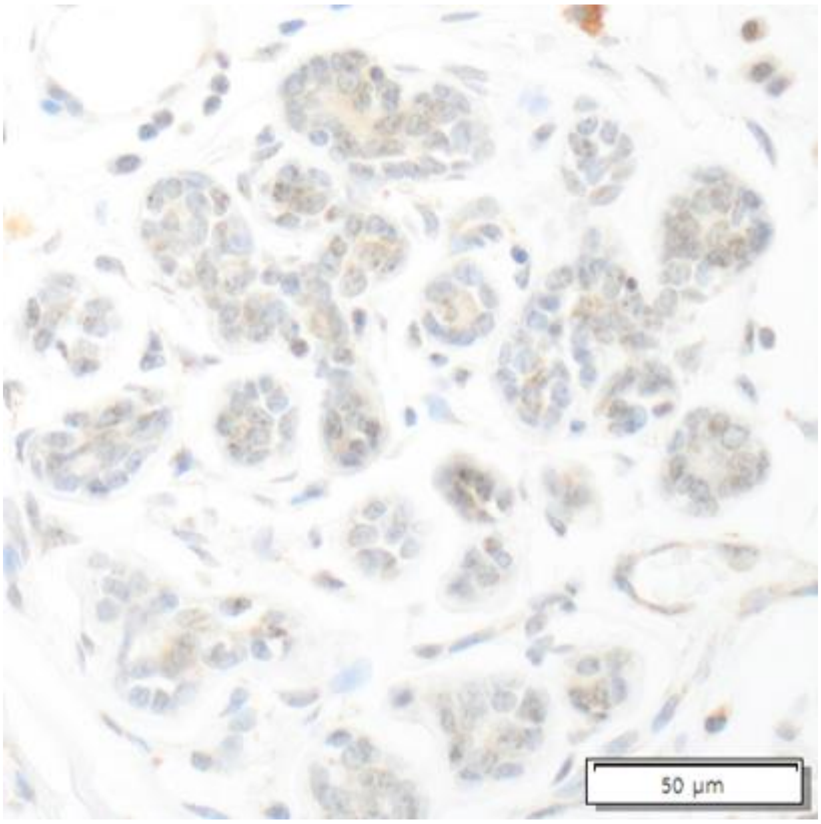

Figure S4. Representative photomicrograph of ASAP1 Immunohistochemical (IHC) staining on the normal breast tissue. The non-neoplastic mammary lobules adjacent to the cancer shows negative to weak positivity on ASAP1 staining.

Table S1. Correlations between ASAP1 expression and clinicopathological characteristics in total cases (n = 452)

Parameters	ASAP1 expression		p-value
	High (n = 176)	Low (n = 276)	
Age (years, mean±SD)	52.5±10.6 (31-83)	53±11 (27-83)	0.608*
Size (cm, mean±SD)	2.6±2 (0.3-16)	2.6±1.6 (0.12-11)	0.612*
Histological grade			0.218‡
G1	41 (23.30%)	58 (21.02%)	
G2	69 (39.20%)	131 (47.46%)	
G3	66 (37.5%)	87 (31.52%)	
T stage			0.159‡
T1,2,3	168 (95.45%)	271 (98.19%)	
T4	8 (4.55%)	5 (1.81%)	
N stage			0.664‡
N0	109 (61.93%)	176 (63.77%)	
N1	36 (20.46%)	62 (22.46%)	
N2	16 (9.09%)	22 (7.97%)	
N3	15 (8.52%)	16 (5.8%)	
Distant metastasis			0.492†
Negative	174 (98.86%)	270 (97.83%)	
Positive	2 (1.14%)	6 (2.17%)	
ER status			0.762‡
Negative	69 (39.2%)	103 (37.32%)	
Positive	107 (60.8%)	173 (62.68%)	
HER2 status			0.651‡
Negative	123 (69.89%)	186 (67.39%)	
Positive	53 (30.11%)	90 (32.61%)	
Ki-67 index			0.075‡
Low (≤10%)	101 (57.39%)	178 (64.49%)	
Interm. (>10%, ≤20%)	28 (15.91%)	25 (9.06%)	
High (>20%)	47 (26.7%)	73 (26.45%)	
Molecular subtype			0.258‡
Luminal A-like	79 (44.89%)	108 (39.13%)	
Luminal B-like	28 (15.91%)	65 (23.55%)	
HER2 (non-luminal)	31 (17.61%)	45 (16.31%)	

Triple-negative	38 (21.59%)	58 (21.01%)

*Student's t-test, †Fisher's exact test, ‡Chi-square test,

ER: estrogen receptor, HER2: human epidermal growth factor receptor 2,

ASAP1: ArfGAP with SH3 domain, ankyrin repeat and PH domain 1

Interm.: Intermediate

Table S2. Correlations between ASAP1 expression and clinicopathological characteristics in ER-positive cases (n = 280)

Parameters	ASAP1 expression		p-value
	High (n = 107)	Low (n = 173)	
Age (years, mean±SD)	53.3±11.4 (31-83)	52.7±11.2 (27-83)	0.687*
Size (cm, mean±SD)	2.2±1.7 (0.3-9)	2.4±1.5 (0.12-9.3)	0.343*
Histological grade			0.512‡
G1	37 (34.58%)	50 (28.9%)	
G2	52 (48.6%)	96 (55.49%)	
G3	18 (16.82%)	27 (15.61%)	
T stage			0.638†
T1,2,3	105 (98.13%)	171 (98.84%)	
T4	2 (1.87%)	2 (1.16%)	
N stage			0.99‡
N0	67 (62.62%)	105 (60.69%)	
N1	25 (23.36%)	43 (24.86%)	
N2	8 (7.48%)	13 (7.51%)	
N3	7 (6.54%)	12 (6.94%)	
HER2 status			0.371‡
Negative	85 (79.44%)	128 (73.99%)	
Positive	22 (20.56%)	45 (26.01%)	
Ki-67 index			0.036‡
Low (≤10%)	75 (70.09%)	124 (71.68%)	
Interm. (>10%, ≤20%)	21 (19.63%)	18 (10.4%)	
High (>20%)	11 (10.28%)	31 (17.92%)	

*Student's t-test, †Fisher's exact test, ‡Chi-square test,

ER: estrogen receptor, HER2: human epidermal growth factor receptor 2,

ASAP1: ArfGAP with SH3 domain, ankyrin repeat and PH domain 1

Interm.: Intermediate
